# Exploring the genetic correlation of cardiovascular diseases and mood disorders in the UK Biobank

**DOI:** 10.1017/S2045796023000252

**Published:** 2023-05-10

**Authors:** Chi-Jen Chen, Wan-Yu Liao, Amrita Chattopadhyay, Tzu-Pin Lu

**Affiliations:** 1 Institute of Epidemiology and Preventive Medicine, Department of Public Health, College of Public Health, National Taiwan University, Taipei, Taiwan; 2 Bioinformatics and Biostatistics Core, Center of Genomics and Precision Medicine, Center of Genomics and Precision Medicine, National Taiwan University, Taipei, Taiwan

**Keywords:** bipolar disorder, chronic conditions, depression, linkage disequilibrium, statistics

## Abstract

**Aims:**

Cardiovascular diseases (CVDs) are the leading cause of deaths globally. Mortality and incidence of CVDs are significantly higher in people with mood disorders. About 81.1% of CVD patients were reported with comorbidities in 2019, where the second most common comorbidity was due to major depressive disorder (MDD). This study, therefore, aimed to evaluate the genetic correlation between CVDs and mood disorders by using data from the UK Biobank towards understanding the influence of genetic factors on the comorbidity due to CVDs and mood disorders.

**Methods:**

The UK Biobank database provides genetic and health information from half a million adults, aged 40–69 years, recruited between 2006 and 2010. A total of 117,925 participants and 6,128,294 variants were included for analysis after applying exclusion criteria and quality control steps. This study focused on two CVD phenotypes, two mood disorders and 12 cardiometabolic-related traits to conduct association studies.

**Results:**

The results indicated a significant positive genetic correlation between CVDs and overall mood disorders and MDD specifically, showing substantial genetic overlap. Genetic correlation between CVDs and bipolar disorder was not significant. Furthermore, significant genetic correlation between mood disorders and cardiometabolic traits was also reported.

**Conclusions:**

The results of this study can be used to understand that CVDs and mood disorders share a great deal of genetic liability in individuals of European ancestry.

## Introduction

Cardiovascular diseases (CVDs) and mood disorders are common and complex diseases. CVDs are the leading cause of death globally. An estimated 18.6 million people died from CVDs in 2019, representing 32% of all global deaths. About 90% of CVDs were attributed to ischemic heart disease, cerebrovascular disease, peripheral artery disease and atrial fibrillation (Roth *et al.*, 2020). Furthermore, about 81.1% of CVD patients had comorbidities. The most common comorbidity was hypertension, with a prevalence of 35.8%. The second most common comorbidity was major depressive disorder (MDD), with a prevalence of 24.5% (Tran *et al.*, 2018).

Epidemiological studies have found that the mortality and incidence of CVDs are significantly higher in people with mood disorders. Regarding mortality, people with bipolar disorder were found to have nearly twice the risk of dying from ischemic heart disease and stroke compared with those without bipolar disorder (Crump *et al.*, 2013). CVDs are also the leading cause of premature death in bipolar disorder patients (Ormel *et al.*, 2007). In addition, patients with MDD had a 2.24-fold odds of dying 2 years after the diagnosis of CVD compared to those without symptoms of MDD (Barth *et al.*, 2004). Regarding incidence, a large meta-analysis found that patients with severe mental illness had a 1.78-fold hazard ratio of CVD, compared to those without severe mental illness (Correll *et al.*, 2017). According to an analysis of the National Health Insurance Research Database of Taiwan, adults aged 20 to 44 with MDD had a 1.50-fold relative risk of developing ischemic heart disease compared to those without MDD. This relative risk was 3.45-fold in patients with a bipolar disorder compared to those without bipolar disorder (Huang *et al.*, 2009). All the aforementioned epidemiological studies, therefore, demonstrated that mood disorders are highly correlated with CVDs. Notably, higher prevalence of CVDs was observed in patients with mood disorders, and conversely, patients with CVDs also tended to develop mood disorders. In summary, these results suggested that there might be a bidirectional association between CVDs and mood disorders and that mood disorders are potentially critical factors determining the prognosis of CVDs.

In genetic studies, both CVDs and mood disorders have exhibited high heritability. For example, the heritability of coronary artery disease (CAD) was 30–60%, that of MDD was 31–42% and that of bipolar disorder was 85–89% (Marenberg *et al.*, 1994; McGuffin *et al.*, 2003; Sullivan *et al.*, 2000). More and more genome-wide association studies (GWASs) have been published in the past decade to dissect the genetic variants in CVDs and mood disorders. For instance, *CACNA1C* is an important gene in the transportation of calcium ions and is associated with the increasing risk of arrhythmia, and the A allele of the single nucleotide polymorphism (SNP) rs1006737 in *CACNA1C* was correlated to a higher risk of bipolar disorder (Ferreira *et al.*, 2008; Napolitano *et al.*, 2012). Furthermore, the genes associated with the phenotypes of various cardiometabolic traits also contributed to the risk of developing CVD (Grallert *et al.*, 2012; McCarthy *et al.*, 2004; van Setten *et al.*, 2013). Several GWASs found that abnormalities in genes encoding ion channel proteins such as SCN5A, CACNA1C and KCNQ2 are not only associated with inherited arrhythmia syndrome (Chen *et al.*, 1998; Juang *et al.*, 2015; Napolitano *et al.*, 2012) but also with bipolar disorder (Ament *et al.*, 2015; Psychiatric GWAS Consortium Bipolar Disorder Working Group, 2011). One additional study showed that the interaction of potassium channel genes *ANK3* and *KCNQ2* were also related to bipolar disorder (Judy *et al.*, 2013). In summary, these studies suggest that certain genetic factors contributing to CVD may also be associated with mood disorders.

Prior studies have demonstrated various traits such as neuroticism, cognitive function and loneliness to play an important role in severe mental disorders and CVDs. A particular study reported neuroticism to have a significant positive genetic correlation with MDD but none with CAD. However, a higher polygenic risk score (PRS) for bipolar disorder, MDD and CAD was associated with higher levels of neuroticism (Gale *et al.*, 2016). Another study showed that a pattern of cognitive functions had a negative genetic correlation with MDD, bipolar disorder and ischemic stroke. In addition, a higher PRS for bipolar disorder, MDD and ischemic stroke was associated with lower levels of a pattern of cognitive functions (Hagenaars *et al.*, 2016). Rødevand *et al.* (2021) found loneliness to have a significant positive genetic correlation with major depression and schizophrenia but not with bipolar disorder. In addition, CVD risk factors, including body mass index (BMI), type 2 diabetes mellitus and coronary heart disease, were significantly positively genetically correlated with loneliness (Rødevand *et al.*, 2021). Furthermore, Dennis *et al.* (2021) performed a phenome-wide association study with a PRS and a multivariable model to explain MDD and loneliness to be related to CAD risk. The results further showed that the PRS of loneliness was significantly associated with mood disorder, depression, ischemic heart disease and coronary atherosclerosis. In minimally adjusted models, the risk of CAD increased with a rise in the standard deviation of the polygenic scores for MDD and loneliness (Dennis *et al.*, 2021).

Mendelian randomization (MR) analysis estimates the causal effect of the exposure on the outcome under study, using genetic instrument variables (e.g., SNP). Some prior studies utilized MR and demonstrated that MDD conferred a causal effect on the risk of cardiovascular conditions, while no reverse causation of cardiovascular condition towards risk of incidence of MDD was observed (Li *et al.*, 2022; Lu *et al.*, 2021; Zhang *et al.*, 2021). Lu *et al.* (2021) showed that genetically instrumented depression was associated with a higher risk of both CAD (odds ratio [OR] = 1.14; 95% confidence interval [CI] = 1.06–1.24; *P* = 1.0 × 10^−3^) and myocardial infarction (OR = 1.21; 95% CI = 1.11–1.33; *P* = 4.8 × 10^−5^). A multivariable MR analysis also presented robust and consistent results (Lu *et al.*, 2021). Li *et al.* (2022) showed that depression had a causal effect on a higher risk of both CAD (OR = 1.099; 95% CI = 1.031–1.170; *P* = 0.004) and myocardial infarction (OR = 1.146; 95% CI = 1.070–1.228; *P* = 1.05 × 10^−4^) using 93 genetic instruments, which was confirmed via sensitivity analyses using 34 genetic instruments (Li *et al.*, 2022).

Overall, epidemiological studies showed that patients with CVDs had a relatively high risk of developing mood disorders, while patients with mood disorders also had a relatively high risk of developing CVDs. The results of previous GWASs also identified several important dysregulated genes that were common to both CVDs and mood disorders, indicating that genetic pleiotropy may play an essential role in the comorbidity of these two complex diseases. To further elucidate the association, this study aimed to evaluate the genetic correlation between CVDs and mood disorders by using data from the UK Biobank to understand the influence of genetic factors on the comorbidity of these two diseases.

## Methods

### Study population

**Figure 1.**
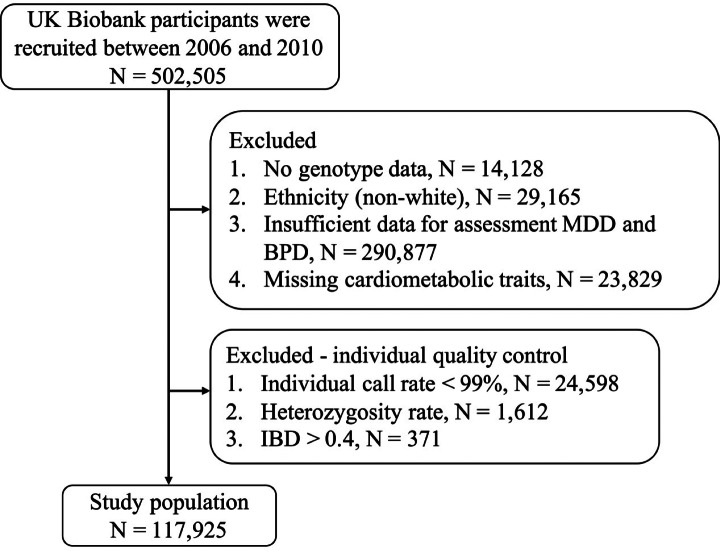
Flow chart. MDD, major depressive disorder; BPD, bipolar disorder; IBD, identical-by-descent.

The UK Biobank database provided genetic and health information from half a million adults aged 40–69 years at study entry (Biobank UK, 2007). Participants were recruited between 2006 and 2010 via interview to collect baseline measurements at one of 22 UK Biobank centres. Extensive questionnaire data, physical measurements and biological samples were collected at recruitment. All participants were followed up for health conditions through linkage to national electronic health-related records. For this analysis, we excluded patients with the following characteristics: (1) no genotype data, (2) non-White ancestry, (3) insufficient data for assessment of MDD and bipolar disorder and (4) missing data on cardiometabolic traits (Fig. 1). Although the UK population includes people of various genetic ancestries, the use of Whites only was designed to avoid complex genetics that might influence the association study results while maintaining the largest possible dataset. The dataset analyzed in this study was retrieved from the UK Biobank. All samples were deidentified, and thus no approval from institutional review board was required.

DNA from the participants’ blood samples were extracted for sequencing using the Affymetrix UK BiLEVE Axiom Array and UK Biobank Axiom Array to obtain genotype information. In order to increase the SNP coverage rate, genotype imputation was conducted to predict unsequenced variants. For the GWAS, high quality of samples and SNPs were ensured to avoid population stratification and inaccurate genotype imputation. Samples with (1) individual call rate <99%, (2) extremely large or small heterozygosity rate and (3) identity-by-descent status >0.4 were excluded (Fig. 1). SNPs with call rate <95%, minor allele frequency (MAF) <5%, Hardy–Weinberg equilibrium *P* > 10^−6^ were excluded from further analysis.

A total of 117,925 participants were identified and 6,128,294 variants were abstracted for analysis after applying the above exclusion criteria and quality control steps. This study focused on two CVD phenotypes (general CVD and arrhythmia), two mood disorders (bipolar disorder and MDD) and 12 cardiometabolic-related traits (diabetes mellitus, hypertension, weight, BMI, body fat percentage, waist–hip ratio (WHR), cholesterol, triglycerides, high-density lipoprotein (HDL), low-density lipoprotein (LDL), systolic blood pressure (SBP) and diastolic blood pressure (DBP) to conduct an association study. The detailed definition of each phenotype, disorder and trait is included in Table S1.

### Genome-wide association study

All phenotypes were subjected to a GWAS after quality control. Using an additive model, we encoded each variant according to the number of minor alleles it contained (0, 1 or 2). We used linear regression to calculate the effects of the allele frequency on the continuous variables (e.g., weight, BMI and other clinical variables). We used logistic regression to calculate the effects of the allele frequency on the categorical variables (e.g., disease phenotypes such as CVD, MDD and diabetes mellitus, encoded as yes/no). We used principal components analysis to check for population stratification and conducted regression analysis adjusted by age and principal components 1–20. The threshold for genome-wide significance was *P* < 5 × 10^−8^. The analysis was conducted in PLINK v2.0 (https://www.cog-genomics.org/plink2).

### Linkage disequilibrium score regression

When using GWAS to evaluate genetic loci, the effect size of most SNPs in complex diseases or phenotypes is usually very small, and only a few genetic loci are found to reach the pre-specified significance level in the research setting. One interpretation of this is that these diseases or phenotypes are co-regulated by multiple loci. Therefore, when evaluating the genetic correlation between diseases and phenotypes, we need to include many gene loci, regardless of whether they exhibit genome-wide significance in the association analysis. This was done using linkage disequilibrium score (LDSC) regression, developed by Bulik-Sullivan *et al.* in 2015, which uses the test statistic of loci in the GWAS to create an LDSC for conducting regression analysis (Bulik-Sullivan *et al.*, 2015). By considering the effects of SNPs and genetic structure, we calculated the heritability of each phenotype and evaluated the genetic correlations between CVD and mood disorders.

This study used the 1000 Genomes Project with European ancestry as a reference panel for LDSCs. The major histocompatibility complex (MHC) on chromosome 6 and gene loci with MAF < 0.01 were excluded. The linkage disequilibrium (LD) in the MHC region is complex, leading to inconsistent LDSCs in this locus. Loci with MAF < 0.01 were excluded because low MAFs can cause extreme test statistics that induce bias into the evaluation of LDSCs.

Heritability (*h*^2^) is defined as the proportion of the variation in a phenotype that can be explained by the GWAS variant and ranges from 0 to 1. If the heritability is equal to 0, the variant does not explain the phenotype; if it is equal to 1, the variant determined the phenotype. The regression coefficient (*r*_g_) estimated by LDSC is the genetic covariance. The genetic correlation of two phenotypes (e.g., CVD and mood disorder) can be obtained by dividing the genetic covariance by the respective heritability of the two phenotypes and then taking the square root. The range of genetic correlation is −1 to 1, where −1 represents a negative relationship and 1 represents a positive relationship. The threshold for statistical significance was *P* < 0.05. The above analysis was conducted using the Python package of ldsc v1.0.1 (https://github.com/bulik/ldsc).

## Results

After application of the exclusion criteria to the UK Biobank database (*n* = 357,999) and of the quality control steps for individual samples (*n* = 26,581), 117,925 participants with qualifying genetic data remained (Fig. 1). After SNP quality control, we removed 3,436,412 variants by three criteria, and 6,128,294 variants remained. Baseline characteristics of this study population are demonstrated in Table 1: 63,154 (53.55%) females, 54,771 (46.45%) males, average age 57.21 and average height 169.04 cm. Table 1 also presents CVD phenotypes including CVDs (*n* = 13,153 [11.15%]) and arrhythmia (*n* = 5,870 [4.98%]), as well as mood disorder phenotypes including MDD (*n* = 22,597 [19.16%]) and bipolar disorder (*n* = 1,024 [0.87%]). Two cardiometabolic traits were categorical, including diabetes mellitus (*n* = 6,371 [5.40%]) and hypertension (*n* = 26,344 [22.34%]). For the 10 continuous cardiometabolic traits, the average values were weight 78.46 kg, BMI 27.38 kg/m^2^, body fat percentage 31.46%, WHR 0.87 cm, cholesterol 5.73 mg/dL, triglycerides 1.73 mg/dL, HDL 1.47 mg/dL, LDL 3.57 mg/dL, SBP 140.88 mmHg and DBP 82.23 mmHg.

**Table 1.** Demographic and clinical characteristics

	Study population (*N* = 117,925)			
Phenotypes	No./mean	%/SD	Median	IQR
*Basic*				
Gender				
Female	63,154	53.55	–	–
Male	54,771	46.45	–	–
Age	57.21	8.02	59	13
Height	169.04	9.23	169	14
*CVD phenotypes*				
CVDs	13,153	11.15	–	–
Arrhythmia	5,870	4.98	–	–
*Cardiometabolic traits*				
Diabetes mellitus	6,371	5.40	–	–
Hypertension	26,344	22.34	–	–
Weight	78.46	15.89	76.9	21.2
BMI	27.38	4.72	26.7	5.8
Body fat percentage	31.46	8.51	31	12.5
WHR	0.87	0.09	0.88	0.13
Cholesterol (total)	5.73	1.15	5.69	1.52
Triglycerides	1.73	1.00	1.48	1.07
HDL	1.47	0.39	1.42	0.51
LDL	3.57	0.87	3.53	1.18
Systolic blood pressure	140.88	19.74	139	26
Diastolic blood pressure	82.23	10.64	82	14
*Mood disorder phenotypes*				
MDD	22,597	19.16	–	–
Bipolar disorder	1,024	0.87	–	–
Mood disorder	23,621	20.03	–	–

IQR, interquartile range

**Figure 2.**
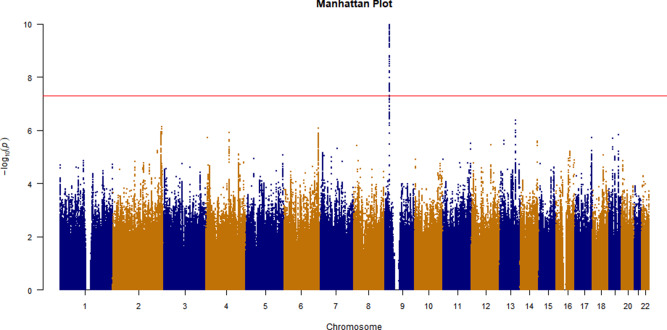
Manhattan plot of GWAS results for CVDs. Red line is significant level *P* < 5 × 10^−8^.

**Figure 3.**
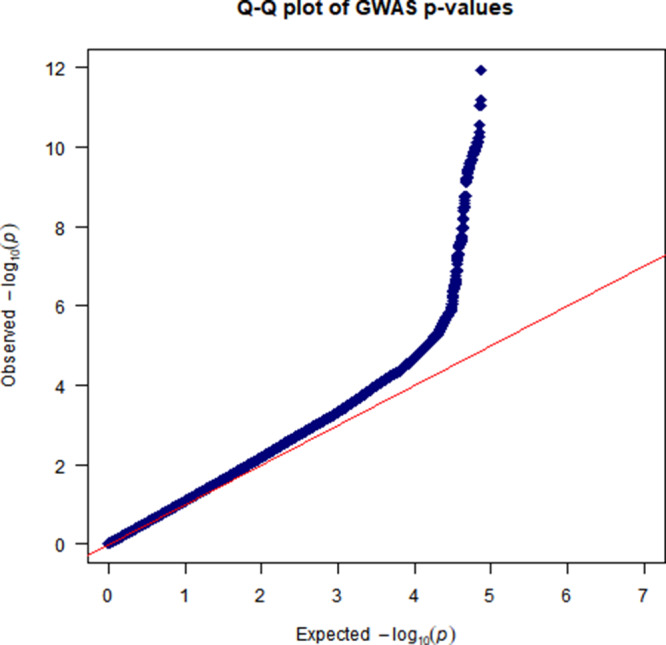
QQ plot of GWAS results for CVDs.

**Figure 4.**
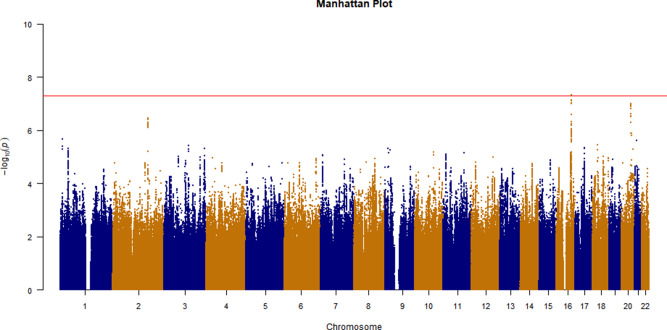
Manhattan plot of GWAS results for mood disorders. Red line is significant level *P* < 5 × 10^−8^.

**Figure 5.**
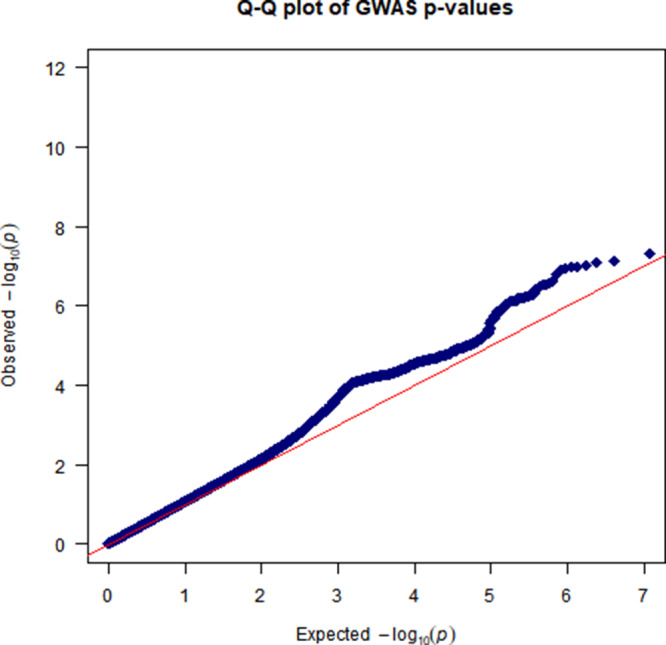
QQ plot of GWAS results for mood disorders.

The CVD GWAS results are summarized in Fig. 2 (Manhattan plot), Fig. 3 (QQ plot) and Table S2 (heritability estimates). Overall, the GWAS data showed moderate deviation in the test statistics compared to the null value (*λ*_GC_ = 1.0988). This deviation was insignificant in the context of this sample size. LDSC regression indicated that the deviation from null was due to polygenic structure, with *h*^2^_SNP_ accounting for ∼2% of the overall variance in CVD (*h*^2^_SNP_ = 0.0185 [SE 0.0036]) rather than inflation due to unconstrained population structure (LD regression intercept = 1.0472 [SE 0.0065]). We observed one independent genomic locus exhibiting genome-wide significant associations with CVD on chromosome 9 (Fig. 2) and four loci associated with arrhythmia on chromosomes 1, 4, 12 and 16 (Figures S1–S2). In addition, we observed three probable independent genomic loci associated with a mood disorder (Figs. 4–5) on chromosomes 2, 16 and 20. Additional results of the GWAS for mood disorders are presented in Figures S3–S6.

We found strong genetic correlation between CVDs and both mood disorders overall (*r*_g_ = 0.5519, SE = 0.1482, *P* = 0.0002) and MDD specifically (*r*_g_ = 0.5620, SE = 0.1636, *P* = 0.0006; Table 2). We did not find significant genetic correlation between CVDs and bipolar disorder (*r*_g_ = 0.3411, SE = 0.2767, *P* = 0.2177; Table 2). Furthermore, this study assessed arrhythmia separately from CVD because CVD encompasses all diseases of heart and blood vessels, while arrhythmia pertains to heart rhythm disorders. Our findings indicate that arrhythmia has genetic causes (Figures S1–S2). However, we did not find significant genetic correlation between arrhythmia and mood disorders (*r*_g_ = 0.0778, SE = 0.1441, *P* = 0.5895), MDD (*r*_g_ = 0.0708, SE = 0.1505, *P* = 0.6379) or bipolar disorder (*r*_g_ = 0.1713, SE = 0.2742, *P* = 0.5322; Table 2).

**Table 2.** Genetic correlations between CVDs and mood disorders

	MDD + BPD				MDD				BPD			
Phenotype	*r*_g_	SE	*P*	*N*	*r*_g_	SE	*P*	*N*	*r*_g_	SE	*P*	*N*
CVD	0.5519	0.1482	0.0002	2,764	0.5620	0.1636	0.0006	2,626	0.3411	0.2767	0.2177	138
Arrhythmia	0.0778	0.1441	0.5895	1,109	0.0708	0.1505	0.6379	1,050	0.1713	0.2742	0.5322	59

BPD, bipolar disorder

Finally, we conducted analyses of the genetic correlation of the 12 cardiometabolic traits (diabetes mellitus, hypertension, weight, BMI, body fat percentage, WHR, total cholesterol, triglycerides, HDL, LDL, SBP and DBP) with CVDs and mood disorders. The results are presented in Table 3. We found strong positive genetic correlations between CVDs and diabetes mellitus (*r*_g_ = 0.6916, SE = 0.1101, *P* = 3.41 × 10^−10^), hypertension (*r*_g_ = 0.9072, SE = 0.0757, *P* = 4.52 × 10^−33^), BMI (*r*_g_ = 0.5474, SE = 0.0651, *P* = 4.05 × 10^−17^) and WHR (*r*_g_ = 0.6252, SE = 0.0733, *P* = 1.50 × 10^−17^). We found moderate genetic correlations between CVDs and weight (*r*_g_ = 0.3613, SE = 0.0601, *P* = 1.79 × 10^−9^), body fat percentage (*r*_g_ = 0.4970, SE = 0.0674, *P* = 1.72 × 10^−13^), triglycerides (*r*_g_ = 0.3969, SE = 0.0723, *P* = 4.09 × 10^−8^), SBP (*r*_g_ = 0.4631, SE = 0.0736, *P* = 3.16 × 10^−10^) and DBP (*r*_g_ = 0.3292, SE = 0.0839, *P* = 8.76 × 10^−5^). However, we found a negative genetic correlation between CVDs and HDL (*r*_g_ = −0.4754, SE = 0.0914, *P* = 1.96 × 10^−7^), along with small, negative but non-significant correlations between CVDs and total cholesterol (*r*_g_ = −0.1821, SE = 0.1107, *P* = 0.0997) and LDL (*r*_g_ = −0.1127, SE = 0.1191, *P* = 0.3441). Furthermore, mood disorders were also identified as having genetic correlation with seven cardiometabolic traits: diabetes mellitus (*r*_g_ = 0.4566, SE = 0.1438, *P* = 0.0015) and hypertension (*r*_g_ = 0.3732, SE = 0.0915, *P* = 4.58 × 10^−5^) had moderate correlations; BMI (*r*_g_ = 0.2320, SE = 0.0654, *P* = 0.0004), body fat percentage (*r*_g_ = 0.1713, SE = 0.0674, *P* = 0.0111), WHR (*r*_g_ = 0.2548, SE = 0.0755, *P* = 0.0007) and triglycerides (*r*_g_ = 0.2450, SE = 0.0644, *P* = 0.0002) had small correlations. In addition, HDL (*r*_g_ = −0.1625, SE = 0.0723, *P* = 0.0245) had a significant negative correlation. Additional results of the genetic correlations of the 12 cardiometabolic traits with arrhythmia and MDD are presented in Table S3.

**Table 3.** Genetic correlations between CVDs, mood disorders and 12 cardiometabolic traits

	CVD			Mood disorder		
Phenotype	*r*_g_	SE	*P*	*r*_g_	SE	*P*
Diabetes mellitus	0.6916	0.1101	3.41 × 10^−10^	0.4566	0.1438	0.0015
Hypertension	0.9072	0.0757	4.52 × 10^−33^	0.3732	0.0915	4.58 × 10^−5^
Weight	0.3613	0.0601	1.79 × 10^−9^	0.1115	0.0616	0.0702
Body mass index	0.5474	0.0651	4.05 × 10^−17^	0.2320	0.0654	0.0004
Body fat percentage	0.4970	0.0674	1.72 × 10^−13^	0.1713	0.0674	0.0111
WHR	0.6252	0.0733	1.50 × 10^−17^	0.2548	0.0755	0.0007
Total cholesterol	−0.1821	0.1107	0.0997	0.0485	0.0784	0.5361
Triglycerides	0.3969	0.0723	4.09 × 10^−8^	0.2450	0.0664	0.0002
HDL	−0.4754	0.0914	1.96 × 10^−7^	−0.1625	0.0723	0.0245
LDL	−0.1127	0.1191	0.3441	0.0626	0.0834	0.4529
Systolic blood pressure	0.4631	0.0736	3.16 × 10^−10^	−0.0271	0.0727	0.7094
Diastolic blood pressure	0.3292	0.0839	8.76 × 10^−5^	−0.0024	0.0664	0.9706

## Discussion

In this study, we have identified one independent locus associated with CVD (rs4977756, *P* = 1.00 × 10^−10^) and three independent loci associated with mood disorders with suggestive significance (rs3769927, *P* = 3.51 × 10^−7^; rs3852786, *P* = 4.75 × 10^−8^ and 20:44555775_GC_G, *P* = 1.03 × 10^−7^). We also identified an SNP-based heritability estimate for CVDs of ∼2% and mood disorders of ∼2%. There was a strong genetic correlation between CVDs and mood disorders, indicating substantial genetic overlap between CVDs and susceptibility to mood disorders. Furthermore, there was also a significant genetic correlation between mood disorders and cardiometabolic traits such as diabetes mellitus, hypertension, BMI, body fat percentage, WHR, triglycerides and HDL. Thus, we observed a genetic overlap between mood disorders and both CVDs and cardiometabolic traits.

Our findings agree well with previous research. A study published in 2018 by Wong *et al.* used LDSC regression to assess cardiometabolic traits in patients with mood disorders, using summary statistics from the Psychiatric Genomics Consortium (PCG) (Wong *et al.*, 2019). The results found a positive genetic correlation between depressive symptoms and CAD, body fat percentage, triglycerides, and WHR. A negative genetic correlation was found between depressive symptoms and HDL (Wong *et al.*, 2019). In addition, a study published in 2020 by Hagenaars *et al.* used LDSC regression to assess cardiometabolic traits in patients with MDD. They used summary statistics from multiple databases from the Psychiatric Genomics Consortium MDD working group (PCG-MDD), Generation Scotland: The Scottish Family Health Study (GS:SFHS) and the UK Biobank. The results found a positive genetic correlation between MDD and BMI, CAD and type 2 diabetes (Hagenaars *et al.*, 2020).

We further assessed the genetic correlation between CVDs, mood disorders and cardiometabolic traits. Our results showed that CVDs were positively and statistically significantly correlated with body weight, SBP and DBP. However, this phenomenon has not been observed in mood disorders. An association between hypertension and depressive symptoms has been found in past studies, but no association has been found between blood pressure and depressive symptoms (López-León *et al.*, 2010). The current study observed a non-significant negative genetic correlation between total cholesterol, LDL and CVDs. However, total cholesterol and LDL showed a non-significant positive genetic correlation in mood disorders. High total cholesterol or LDL was found in past studies to predict CVD, but it only showed association and did not explain causation. Few studies have adjusted for other CVD-promoting factors, such as mental stress. Mental stress raises total cholesterol probably because cholesterol is necessary for the production of cortisol and other steroid stress hormones, and mental stress may contribute to CVDs by increasing the production of epinephrine and norepinephrine, which can lead to hypertension and hypercoagulation (Ravnskov *et al.*, 2018).

This study used questionnaire data to define mood disorders similar to the approach adopted by Smith *et al.* (2013) Previous studies have utilized summary statistics from UK Biobank, 23andMe, PGC-MDD and GS:SFHS (Hagenaars *et al.*, 2020; Howard *et al.*, 2019, 2018; Li *et al.*, 2022). Table S4 provides a comparison of the phenotypic definitions as were used in various prior studies. The definitions of mood disorders that were used in prior studies were more complex and were based on questionnaire data, diagnostic codes (ICD-10) in electronic health records and usage of antipsychotic drugs. Different studies used different definitions. For example, depressive disorder was determined in patients via questionnaire with questions such as “Have you ever seen a family doctor or a psychiatrist because of nervous anxiety, tension or depression?”; however, exclusions were applied to patients with bipolar disorder, schizophrenia or personality disorders and those who were on antipsychotic medication (Howard *et al.*, 2019, 2018). Some studies used only questionnaire data, while others used questionnaire data, diagnostic codes and medications to define depression. Hence, the definitions varied significantly among studies. On the other hand, the definition of CVD was more straightforward using only the diagnostic code (ICD-10). CVDs included coronary heart disease, heart failure, cerebrovascular disease and peripheral artery disease in this study. Previous studies by the CARDIoGRAMplusC4D Consortium focused on CAD (Hagenaars *et al.*, 2020; Howard *et al.*, 2019, 2018; Li *et al.*, 2022). This study included additional cardiovascular conditions to maximize the sample size.

Table S5 demonstrates the comparison of lambda values and LDSC intercepts reported in this study with those that were reported in previous studies. The *λ*_GC_ value was 1.09 for mood disorders and 1.099 for CVDs in this study, indicating a negligible population stratification effect. *λ*_GC_ as reported by prior studies were 1.3238 for broad depression and 1.63 for depression, both of which were significantly higher than 1, indicating existence of batch effects due to meta-analysis from different studies (Hagenaars *et al.*, 2020; Howard *et al.*, 2019, 2018; Li *et al.*, 2022). As for LDSC intercepts, this study reported 1.03 for mood disorders and 1.05 for CVDs, which was close to 1, indicating that the influence of confounding factors was small. Prior studies, broad depression was 1.0079 and depression was 1.015, which was also closer to 1, indicating that confounding factors had less influence. However, 0.8881 for the CAD was far from 1, indicating that it may be affected by confounding factors (Hagenaars *et al.*, 2020; Howard *et al.*, 2019, 2018; Li *et al.*, 2022).

Most past studies have investigated the genetic correlations of cardiometabolic traits (e.g., BMI and diabetes mellitus), CAD, stroke and depression separately but not in an integrated way (Bahrami *et al.*, 2020; Hagenaars *et al.*, 2020; López-León *et al.*, 2010; Wong *et al.*, 2019). Our study defined CVD broadly to include coronary heart disease (i.e., CAD or ischemic heart disease), heart failure, cerebrovascular disease and peripheral artery disease. A broad definition allows the inclusion of a large number of cases, which is needed to maximize the statistical power. While broadly defining CVD has its advantages, it also has disadvantages. One disadvantage may be that there are in actual differences between these diseases in terms of their risk factors. For example, risk factors such as smoking and diabetes are more strongly associated with coronary heart disease than stroke (Matsunaga *et al.*, 2017). Therefore, further research will be needed to determine the genetic correlations between mood disorders and the subtypes of CVD. Subsequent studies will need to use MR to explore the causal relationships.

This study used the UK Biobank as the sole data source, and thus only genetic correlations in individuals of European ancestry could be explored. In a past study, individuals of East Asian ancestry have been found to have negative genetic correlations with CAD, BMI and type 2 diabetes (Giannakopoulou *et al.*, 2021), contrary to the results observed in this study. The previous study noted that the negative correlation might be due to recruitment strategies or social factors. However, whether the same phenomenon would be observed in individuals of other ancestry requires further verification, so for now, all research results on this topic must be interpreted in light of the study population.

## Conclusions

This study found positive genetic correlations between CVDs and mood disorders in individuals of European ancestry. This means that CVDs and mood disorders share a great deal of genetic liability. Genetic correlations between mood disorders and cardiometabolic traits were also found. However, further studies will be needed to explore the causal relationship between CVDs and mood disorders.

## Data Availability

The dataset used in this study can be accessed from the UK Biobank (https://www.ukbiobank.ac.uk/enable-your-research/apply-for-access). Computing code can be available through the corresponding author request.

## Supplementary material

For supplementary material accompanying this paper visit http://doi.org/10.1017/S2045796023000252. click here to view supplementary material
