# Supplementary material for: Exploring the genetic correlation of cardiovascular diseases and mood disorders in the UK Biobank
Source: Epidemiol Psychiatr Sci. 2023 May 10;32:e31. doi: 10.1017/S2045796023000252 (PMC10227537; doi:10.1017/S2045796023000252)
Supplement: Supplementary file 1 [file S2045796023000252sup001.docx]

*Supplementary data*

**Exploring the genetic correlation of cardiovascular diseases and mood disorders in the UK biobank**

Chi-Jen Chen^1^, Wan-Yu Liao^1^, Amrita Chattopadhyay^2^, Tzu-Pin Lu^1,^*

The supplemental data included the following information:

1. Supplementary Figure S1-S6
2. Supplementary Table S1-S5

**
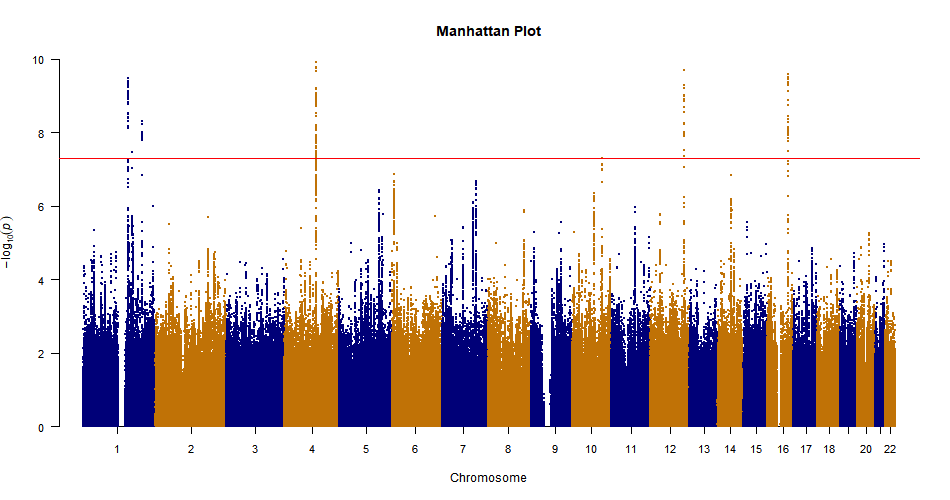
**

**Figure S1. Manhattan plot of GWAS results for arrhythmia.** Red line is significant level *P* < 5x10^-8^

**
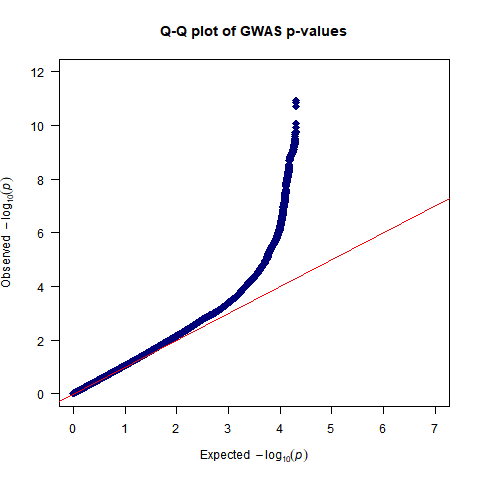
**

**Figure S2. QQ plot of GWAS results for arrhythmia**

**
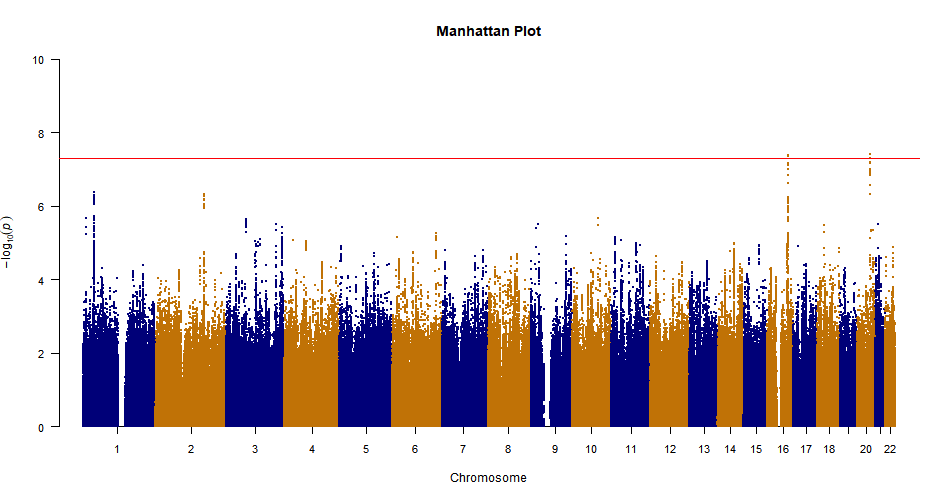
**

**Figure S3. Manhattan plot of GWAS results for major depressive disorder.** Red line is significant level *P* < 5x10^-8^

**
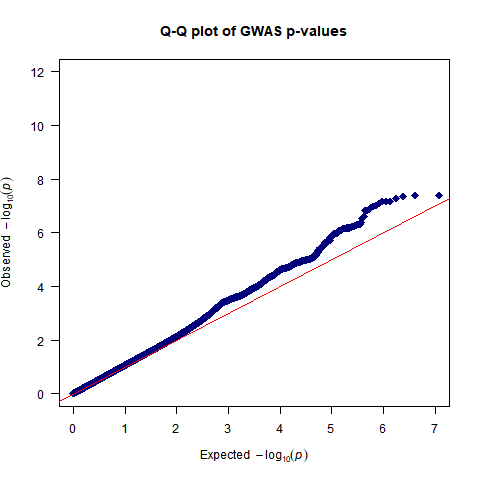
**

**Figure S4. QQ plot of GWAS results for major depressive disorder**

**
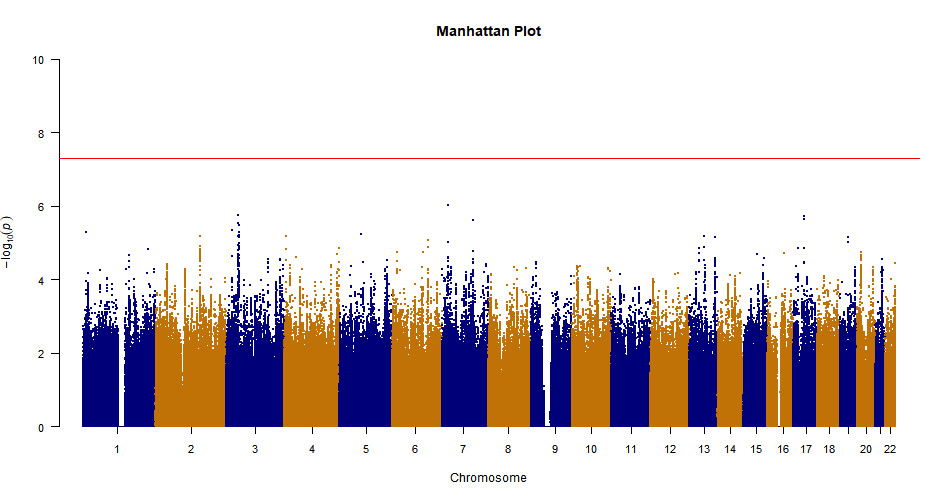
**

**Figure S5. Manhattan plot of GWAS results for bipolar disorder.** Red line is significant level *P* < 5x10^-8^

**
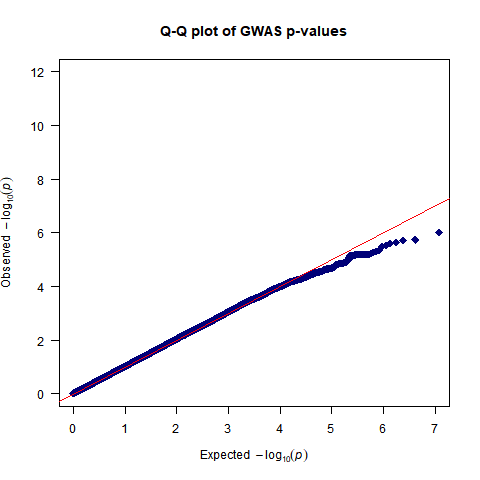
**

**Figure S6. QQ plot of GWAS results for bipolar disorder**

Table S1. Definition of phenotypes

| No. | Phenotypes | Definition |
| --- | --- | --- |
| 1 | Cardiovascular disease (CVD) | In-patient records include the following diagnosis. Coronary heart disease ICD-10: I20, I21, I22, I23, I24, I25; Heart failure   ICD-10: I50; Cerebrovascular disease  ICD-10: I60, I61, I62, I63, I64, I65, I66, I67, I68, I69.0, I69.1, I69.2, I69.3, I69.4, I69.8, G45, G46; Peripheral artery disease  ICD-10: I71, I72, I74, I73.9, I70.21. |
| 2 | Arrhythmia | In-patient records include the following diagnosis. Atrial Fibrillation/Flutter ICD-10: I48, I48.1, I48.2, I48.3, I48.4, I48.9; Ventricular Arrhythmias ICD-10: I47.0, I47.2, I49.0, I46.0, I46.1, I46.9; Bradyarrhythmia ICD-10: I44.0, I44.1, I44.2, I44.3, I44.5, I49.5 |
| 3 | Mood disorder | Either major depressive disorder or bipolar disorder. |
| 4 | Major depressive disorder (MDD) | Single probable episode of major depression: EITHER: 4598 Ever depressed/down for a whole week, plus 4609 At least two weeks duration, plus 4620 Only one episode, plus 2090 Ever seen a GP or 2100 a psychiatrist for nerves, anxiety, depression OR: 4631 Ever anhedonic (unenthusiasm/uninterest) for a whole week, plus 5375 At least two weeks, plus 5386 Only one episode, plus 2090 Ever seen a GP or 2100 a psychiatrist for nerves, anxiety, depression Probable recurrent major depression (moderate): EITHER: 4598 Ever depressed/down for a whole week, plus 4609 At least two weeks duration, plus 4620 At least two episodes, plus 2090 Ever seen a GP (but not a psychiatrist) for nerves, anxiety, depression OR: 4631 Ever anhedonic (unenthusiasm/uninterest) for a whole week, plus 5375 At least two weeks, plus 5386 At least two episodes, plus 2090 Ever seen a GP (but not a psychiatrist) for nerves, anxiety, depression Probable recurrent major depression (severe): EITHER: 4598 Ever depressed/down for a whole week, plus 4609 At least two weeks duration, plus 4620 At least two episodes, plus 2100 Ever seen a psychiatrist for nerves, anxiety, depression OR: 4631 Ever anhedonic (unenthusiasm/uninterest) for a whole week, plus 5375 At least two weeks, plus 5386 At least two episodes, plus 2100 Ever seen a psychiatrist for nerves, anxiety, depression |
| 5 | Bipolar disorder | Probable bipolar disorder (type I): Either: 4642 Ever manic/hyper 2 days or 4653 Ever irritable/argumentative for 2 days, plus At least 3 from 6156.01 (more active), 6156.02 (more talkative), 6156.03 (needed less sleep), and 6156.04 (more creative/more ideas), plus 5663 Duration of a week or more, plus 5674 needed treatment or caused problems at work Probable bipolar disorder (type II): Either: 4642 Ever manic/hyper 2 days or 4653 Ever irritable/argumentative for 2 days, plus At least 3 from 6156.01 (more active), 6156.02 (more talkative), 6156.03 (needed less sleep), and 6156.04 (more creative/more ideas), plus 5663 Duration of a week or more |
| 6 | Diabetes mellitus | In-patient records include the following diagnosis. ICD-10: E08, E09, E10, E11, E13. |
| 7 | Hypertension | In-patient records include the following diagnosis. ICD-10: I10, I11.9, I13.10, I15.0, I15.1, I15.2, I15.8, I15.9, N26.2, I11, I12, I13. |
| 8 | Weight | kg |
| 9 | Body mass index (BMI) | weight (kg) / height (m2) |
| 10 | Body fat percentage | % |
| 11 | Waist-hip ratio (WHR) | cm |
| 12 | Cholesterol | mg/dL |
| 13 | Triglycerides | mg/dL |
| 14 | High-density lipoprotein (HDL) | mg/dL |
| 15 | Low-density lipoprotein (LDL) | mg/dL |
| 16 | Systolic blood pressure (SBP) | mmHg |
| 17 | Diastolic blood pressure (DBP) | mmHg |

Table S2. Heritability

| Phenotype | h^2^ SNP (S.E.) | Lambda GC | LDSC intercept (S.E.) | Mean Chi-sq |
| --- | --- | --- | --- | --- |
| CVD | 0.0185 (0.0036) | 1.0988 | 1.0472 (0.0065) | 1.1007 |
| Arrhythmia | 0.0177 (0.0043) | 1.0557 | 1.0202 (0.0073) | 1.0736 |
| Mood disorder | 0.0190 (0.0038) | 1.0864 | 1.0288 (0.0074) | 1.0877 |
| MDD | 0.0165 (0.0036) | 1.0772 | 1.0272 (0.0074) | 1.0785 |
| Bipolar disorder | 0.0042 (0.0029) | 1.0165 | 1.0028 (0.0060) | 1.0167 |
| Diabetes mellitus | 0.0270 (0.0042) | 1.0926 | 1.0394 (0.0078) | 1.1185 |
| Hypertension | 0.0551 (0.0055) | 1.1973 | 1.0702 (0.0087) | 1.2269 |
| Weight | 0.2005 (0.0104) | 1.4817 | 1.1131 (0.0118) | 1.6376 |
| BMI | 0.1908 (0.0094) | 1.4926 | 1.1101 (0.0114) | 1.6215 |
| Body fat percentage | 0.1734 (0.0091) | 1.4709 | 1.1292 (0.0111) | 1.5937 |
| WHR | 0.1126 (0.0073) | 1.3306 | 1.0969 (0.0098) | 1.4119 |
| Total cholesterol | 0.0950 (0.0146) | 1.1683 | 1.0585 (0.0109) | 1.3154 |
| Triglycerides | 0.1221 (0.0210) | 1.2168 | 1.1058 (0.0114) | 1.4292 |
| HDL | 0.1610 (0.0212) | 1.3306 | 1.1408 (0.0138) | 1.5609 |
| LDL | 0.0862 (0.0156) | 1.1491 | 1.0560 (0.0113) | 1.2852 |
| SBP | 0.0989 (0.0070) | 1.2764 | 1.0936 (0.0094) | 1.3678 |
| DBP | 0.1037 (0.0073) | 1.2899 | 1.0803 (0.0093) | 1.3681 |

SNP, single nucleotide polymorphism; Lambda GC, genomic inflation factor; LDSC, linkage disequilibrium score; CVD, cardiovascular disease; MDD, major depressive disorder; BMI, body mass index; WHR, waist-hip ratio; HDL, high-density lipoprotein; LDL, low-density lipoprotein; SBP, systolic blood pressure; DBP, diastolic blood pressure.

Table S3. Genetic correlations between arrhythmia, MDD and 12 cardiometabolic traits

| Phenotype | Arrhythmia | | |  | MDD | | |
| --- | --- | --- | --- | --- | --- | --- | --- |
|  | r_g_ | S.E. | P |  | r_g_ | S.E. | P |
| Diabetes mellitus | 0.2785 | 0.1213 | 0.0217 |  | 0.4945 | 0.1564 | 0.0016 |
| Hypertension | 0.3893 | 0.0944 | $3.71\times{10}^{-5}$ |  | 0.4049 | 0.1016 | $6.70\times{10}^{-5}$ |
| Weight | 0.3912 | 0.0677 | $7.36\times{10}^{-9}$ |  | 0.1255 | 0.0657 | 0.0561 |
| BMI | 0.2914 | 0.0687 | $2.20\times{10}^{-5}$ |  | 0.2577 | 0.0697 | 0.0002 |
| Body fat percentage | 0.3262 | 0.0722 | $6.28\times{10}^{-6}$ |  | 0.2031 | 0.0716 | 0.0046 |
| WHR | 0.3006 | 0.0792 | 0.0001 |  | 0.2773 | 0.0783 | 0.0004 |
| Total cholesterol | -0.1363 | 0.0856 | 0.1113 |  | 0.0342 | 0.0806 | 0.6711 |
| Triglycerides | 0.1054 | 0.0810 | 0.1932 |  | 0.2548 | 0.0687 | 0.0002 |
| HDL | -0.2507 | 0.0781 | 0.0013 |  | -0.1978 | 0.0756 | 0.0089 |
| LDL | -0.0760 | 0.0927 | 0.4124 |  | 0.0576 | 0.0858 | 0.5025 |
| SBP | 0.1660 | 0.0826 | 0.0444 |  | -0.0294 | 0.0771 | 0.7029 |
| DBP | 0.1594 | 0.0801 | 0.0466 |  | 0.0064 | 0.0697 | 0.9265 |

MDD, major depressive disorder; BMI, body mass index; WHR, waist-hip ratio; HDL, high-density lipoprotein; LDL, low-density lipoprotein; SBP, systolic blood pressure; DBP, diastolic blood pressure.

Table S4. Comparison of the phenotypic definitions between the current study and the previous studies

| Author | Trait 1 | Trait 2 |
| --- | --- | --- |
| This study | **Mood disorder**   - Either major depressive disorder or bipolar disorder by the touchscreen response questionnaire. The details are shown in Supplementary Table S1. | **Cardiovascular diseases**   - Including coronary heart disease, heart failure, cerebrovascular disease, and peripheral artery disease by in-patient records of ICD-10 codes. The details are shown in Supplementary Table S1. |
| Howard *et al.*, 2018 | **Broad depression**   - From touchscreen questionnaire at recruitment: "Have you ever seen a GP/psychiatrist for nerves, anxiety, tension or depression?". - From Hospital Episodes Data from UK bodies (English HES Data, Scottish Morbidity Register, Patient Episode Data). Any primary or secondary diagnosis of ICD-10 Codes for mood disorders (F32–Single Episode Depression, F33–Recurrent Depression, F34–Persistent mood disorders, F38–Other mood disorders and F39–Unspecified mood disorders) | **Coronary artery disease**   - A meta-analysis of 48 GWAS of CAD of CARDIoGRAMplusC4D Consortium. (Nikpay *et al.*, 2015) - Case status was defined by an inclusive diagnosis criterion of CAD (myocardial infarction, acute coronary syndrome, chronic stable angina or coronary stenosis of >50%). |
| Howard *et al.*, 2019 | **Depression**   - This study was collected summary statistics from UK Biobank, 23andMe, and the Psychiatric Genomics Consortium (PGC) MDD working group (PGC-MDD). - (UK Biobank) From touchscreen questionnaire at recruitment: "Have you ever seen a GP/psychiatrist for nerves, anxiety, tension or depression?" Exclusions were applied to participants who were identified with bipolar disorder, schizophrenia, or personality disorder using self-declared data following the approach of Smith, et al. as well as prescriptions for antipsychotic medications. (Smith *et al.*, 2013) - (23andMe) Phenotypic status was based on responses to web-based surveys with individuals that self-reported as having received a clinical diagnosis or treatment for depression classified as cases. - (PGC) Clinically-derived (DSM-IV) phenotypes for MDD. | **Coronary artery disease**   - A meta-analysis of 48 GWAS of CAD of CARDIoGRAMplusC4D Consortium. (Nikpay *et al.*, 2015) - Case status was defined by an inclusive diagnosis criterion of CAD (myocardial infarction, acute coronary syndrome, chronic stable angina or coronary stenosis of >50%). |
| Hagenaars *et al.*, 2020 | **Major depressive disorder**   - This study was performed using data from the Psychiatric Genomics Consortium (PGC) MDD working group (PGC-MDD), Generation Scotland: The Scottish Family Health Study (GS:SFHS), and UK Biobank. - (PGC-MDD) All cases were required to have a lifetime diagnosis of MDD based on international consensus criteria (DSM-IV, ICD-9, or ICD-10). - (GS:SFHS) MDD diagnosis was based on the Structured Clinical Interview for DSM-IV disorders. Participants who answered positively to two mental health screening questions were invited to complete the full SCID to ascertain MDD diagnosis. Cases were further refined through NHS linkage. - (UK Biobank) MDD cases were defined as individuals meeting lifetime criteria for MDD based on questions from the Composite International Diagnostic Interview. Individuals reporting previous self-reported diagnosis of schizophrenia (or other psychosis) or bipolar disorder were excluded as MDD cases. | **Coronary artery disease**   - A meta-analysis of 48 GWAS of CAD of CARDIoGRAMplusC4D Consortium. (Nikpay *et al.*, 2015) - Case status was defined by an inclusive diagnosis criterion of CAD (myocardial infarction, acute coronary syndrome, chronic stable angina or coronary stenosis of >50%). |
| Li *et al.*, 2022 | **Broad depression**   - A GWAS of broad depression (self-reported past help-seeking from general practitioners or psychiatrists for problems with “nerve, anxiety, tension or depression”) conducted in UK Biobank participants. (Howard *et al.*, 2018) | **Myocardial infarction**   - A meta-analysis of 48 GWAS of coronary artery diseases of CARDIoGRAMplusC4D Consortium. Sub-group analysis was done for subjects with reported history of myocardial infarction. (Nikpay *et al.*, 2015) |
|  | **Depression**   - A meta-analysis of three largest GWAS of depression from the three largest studies of different depression phenotypes, including self-reported clinical diagnosis or treatment of depression, self-reported diagnosis together with conventional methods like structured diagnostic interviews, and broad depression. (Howard *et al.*, 2019) | **Atrial fibrillation**   - A meta-analysis of GWAS of atrial fibrillation from 6 studies: (Nielsen *et al.*, 2018) The Nord-Trøndelag Health Study (HUNT), deCODE, the Michigan Genomics Initiative (MGI), DiscovEHR, UK Biobank, and the AFGen Consortium. Cases and controls are mainly defined by ICD codes. |

Table S5. Comparison of lambda and LDSC intercepts between the current study and previous studies

| Author | Trait 1 | | |  | Trait 2 | | | r_g_  (S.E.) |
| --- | --- | --- | --- | --- | --- | --- | --- | --- |
|  | Trait | Lambda GC | LDSC intercept  (S.E.) |  | Trait | Lambda GC | LDSC intercept  (S.E.) |  |
| This study | MD | 1.0864 | 1.0288  (0.0074) |  | CVD | 1.0988 | 1.0472 (0.0065) | 0.5519 (0.1482) |
| Howard *et al.*, 2018 | BD | 1.3238 | 1.0079  (0.0078) |  | CAD | – | 0.8881  (0.0094) | 0.1236  (0.0288) |
| Howard *et al.*, 2019 | Depression | 1.63 | 1.015  (0.011) |  | CAD | – | 0.8881  (0.0094) | 0.1328  (0.0244) |
| Hagenaars *et al.*, 2020 | MDD | – | 1.0096  (0.0094) |  | CAD | – | 0.8881  (0.0094) | 0.121  (0.03) |
| Li *et al.*, 2022 | BD | 1.3238 | 1.0079  (0.0078) |  | MI | – | – | 0.1231 (0.0324) |
|  | BD | 1.3238 | 1.0079  (0.0078) |  | AF | – | – | 0.1258 (0.0272) |
|  | Depression | 1.63 | 1.015  (0.011) |  | MI | – | – | 0.1688 (0.0294) |
|  | Depression | 1.63 | 1.015  (0.011) |  | AF | – | – | 0.1124 (0.0251) |

MD, mood disorder; BD, broad depression; MDD, major depressive disorder; CVD, cardiovascular disease; CAD, coronary artery disease; MI, Myocardial infarction; AF, atrial fibrillation

References

**Hagenaars, S.P., Coleman, J.R.I., Choi, S.W., Gaspar, H., Adams, M.J., Howard, D.M., Hodgson, K., Traylor, M., Air, T.M., Andlauer, T.F.M., Arolt, V., Baune, B.T., Binder, E.B., Blackwood, D.H.R., Boomsma, D.I., Campbell, A., Cearns, M., Czamara, D., Dannlowski, U., Domschke, K., de Geus, E.J.C., Hamilton, S.P., Hayward, C., Hickie, I.B., Hottenga, J.J., Ising, M., Jones, I., Jones, L., Kutalik, Z., Lucae, S., Martin, N.G., Milaneschi, Y., Mueller-Myhsok, B., Owen, M.J., Padmanabhan, S., Penninx, B.W.J.H., Pistis, G., Porteous, D.J., Preisig, M., Ripke, S., Shyn, S.I., Sullivan, P.F., Whitfield, J.B., Wray, N.R., McIntosh, A.M., Deary, I.J., Breen, G., Lewis, C.M.** (2020). Genetic comorbidity between major depression and cardio-metabolic traits, stratified by age at onset of major depression. *American Journal of Medical Genetics. Part B, Neuropsychiatric Genetics: The Official Publication of the International Society of Psychiatric Genetics*, **183**: 309–330.

**Howard, D.M., Adams, M.J., Clarke, T.-K., Hafferty, J.D., Gibson, J., Shirali, M., Coleman, J.R.I., Hagenaars, S.P., Ward, J., Wigmore, E.M., Alloza, C., Shen, X., Barbu, M.C., Xu, E.Y., Whalley, H.C., Marioni, R.E., Porteous, D.J., Davies, G., Deary, I.J., Hemani, G., Berger, K., Teismann, H., Rawal, R., Arolt, V., Baune, B.T., Dannlowski, U., Domschke, K., Tian, C., Hinds, D.A., Trzaskowski, M., Byrne, E.M., Ripke, S., Smith, D.J., Sullivan, P.F., Wray, N.R., Breen, G., Lewis, C.M., McIntosh, A.M.** (2019). Genome-wide meta-analysis of depression identifies 102 independent variants and highlights the importance of the prefrontal brain regions. *Nature neuroscience*, **22**: 343–352.

**Howard, D.M., Adams, M.J., Shirali, M., Clarke, T.-K., Marioni, R.E., Davies, G., Coleman, J.R.I., Alloza, C., Shen, X., Barbu, M.C., Wigmore, E.M., Gibson, J., Hagenaars, S.P., Lewis, C.M., Ward, J., Smith, D.J., Sullivan, P.F., Haley, C.S., Breen, G., Deary, I.J., McIntosh, A.M.** (2018). Genome-wide association study of depression phenotypes in UK Biobank identifies variants in excitatory synaptic pathways. *Nature Communications*, **9**: 1470.

**Li, G.H.-Y., Cheung, C.-L., Chung, A.K.-K., Cheung, B.M.-Y., Wong, I.C.-K., Fok, M.L.Y., Au, P.C.-M., Sham, P.-C.** (2022). Evaluation of bi-directional causal association between depression and cardiovascular diseases: a Mendelian randomization study. *Psychological Medicine*, **52**: 1765–1776.

**Nielsen, J.B., Thorolfsdottir, R.B., Fritsche, L.G., Zhou, W., Skov, M.W., Graham, S.E., Herron, T.J., McCarthy, S., Schmidt, E.M., Sveinbjornsson, G., Surakka, I., Mathis, M.R., Yamazaki, M., Crawford, R.D., Gabrielsen, M.E., Skogholt, A.H., Holmen, O.L., Lin, M., Wolford, B.N., Dey, R., Dalen, H., Sulem, P., Chung, J.H., Backman, J.D., Arnar, D.O., Thorsteinsdottir, U., Baras, A., O’Dushlaine, C., Holst, A.G., Wen, X., Hornsby, W., Dewey, F.E., Boehnke, M., Kheterpal, S., Mukherjee, B., Lee, S., Kang, H.M., Holm, H., Kitzman, J., Shavit, J.A., Jalife, J., Brummett, C.M., Teslovich, T.M., Carey, D.J., Gudbjartsson, D.F., Stefansson, K., Abecasis, G.R., Hveem, K., Willer, C.J.** (2018). Biobank-driven genomic discovery yields new insight into atrial fibrillation biology. *Nature Genetics*, **50**: 1234–1239.

**Nikpay, M., Goel, A., Won, H.-H., Hall, L.M., Willenborg, C., Kanoni, S., Saleheen, D., Kyriakou, T., Nelson, C.P., Hopewell, J.C., Webb, T.R., Zeng, L., Dehghan, A., Alver, M., Armasu, S.M., Auro, K., Bjonnes, A., Chasman, D.I., Chen, S., Ford, I., Franceschini, N., Gieger, C., Grace, C., Gustafsson, S., Huang, Jie, Hwang, S.-J., Kim, Y.K., Kleber, M.E., Lau, K.W., Lu, X., Lu, Y., Lyytikäinen, L.-P., Mihailov, E., Morrison, A.C., Pervjakova, N., Qu, L., Rose, L.M., Salfati, E., Saxena, R., Scholz, M., Smith, A.V., Tikkanen, E., Uitterlinden, A., Yang, X., Zhang, W., Zhao, W., de Andrade, M., de Vries, P.S., van Zuydam, N.R., Anand, S.S., Bertram, L., Beutner, F., Dedoussis, G., Frossard, P., Gauguier, D., Goodall, A.H., Gottesman, O., Haber, M., Han, B.-G., Huang, Jianfeng, Jalilzadeh, S., Kessler, T., König, I.R., Lannfelt, L., Lieb, W., Lind, L., Lindgren, C.M., Lokki, M.-L., Magnusson, P.K., Mallick, N.H., Mehra, N., Meitinger, T., Memon, F.-U.-R., Morris, A.P., Nieminen, M.S., Pedersen, N.L., Peters, A., Rallidis, L.S., Rasheed, A., Samuel, M., Shah, S.H., Sinisalo, J., Stirrups, K.E., Trompet, S., Wang, L., Zaman, K.S., Ardissino, D., Boerwinkle, E., Borecki, I.B., Bottinger, E.P., Buring, J.E., Chambers, J.C., Collins, R., Cupples, L.A., Danesh, J., Demuth, I., Elosua, R., Epstein, S.E., Esko, T., Feitosa, M.F., Franco, O.H., Franzosi, M.G., Granger, C.B., Gu, D., Gudnason, V., Hall, A.S., Hamsten, A., Harris, T.B., Hazen, S.L., Hengstenberg, C., Hofman, A., Ingelsson, E., Iribarren, C., Jukema, J.W., Karhunen, P.J., Kim, B.-J., Kooner, J.S., Kullo, I.J., Lehtimäki, T., Loos, R.J.F., Melander, O., Metspalu, A., März, W., Palmer, C.N., Perola, M., Quertermous, T., Rader, D.J., Ridker, P.M., Ripatti, S., Roberts, R., Salomaa, V., Sanghera, D.K., Schwartz, S.M., Seedorf, U., Stewart, A.F., Stott, D.J., Thiery, J., Zalloua, P.A., O’Donnell, C.J., Reilly, M.P., Assimes, T.L., Thompson, J.R., Erdmann, J., Clarke, R., Watkins, H., Kathiresan, S., McPherson, R., Deloukas, P., Schunkert, H., Samani, N.J., Farrall, M.** (2015). A comprehensive 1,000 Genomes-based genome-wide association meta-analysis of coronary artery disease. *Nature Genetics*, **47**: 1121–1130.

**Smith, D.J., Nicholl, B.I., Cullen, B., Martin, D., Ul-Haq, Z., Evans, J., Gill, J.M.R., Roberts, B., Gallacher, J., Mackay, D., Hotopf, M., Deary, I., Craddock, N., Pell, J.P.** (2013). Prevalence and Characteristics of Probable Major Depression and Bipolar Disorder within UK Biobank: Cross-Sectional Study of 172,751 Participants. *PLOS ONE*, **8**: e75362.
